# Male Red Ornamentation Is Associated with Female Red Sensitivity in Sticklebacks

**DOI:** 10.1371/journal.pone.0025554

**Published:** 2011-09-30

**Authors:** Ingolf P. Rick, Marion Mehlis, Theo C. M. Bakker

**Affiliations:** Institute for Evolutionary Biology and Ecology, University of Bonn, Bonn, Germany; Ecole Normale Supérieure de Lyon, France

## Abstract

Sexual selection theory proposes correlated evolutionary changes in mating preferences and secondary sexual characters based on a positive genetic correlation between preference and the preferred trait. Empirical work has provided support for a genetic covariation between female preference and male attractiveness in several taxa. Here, we study parent and offspring visual traits in threespine sticklebacks, *Gasterosteus aculeatus*. While focusing on the proximate basis of mating preferences, we compare the red breeding coloration of males, which strongly contributes to female choice, with their daughters' red sensitivity measured by optomotor response thresholds. We show that the red color expression of fathers correlates well with their daughters' red sensitivity. Given that a within-population genetic correlation between signal and preference was experimentally confirmed for the red coloration in sticklebacks, our results indicate a proximate mechanism in terms of perceptual sensitivity being involved in the co-evolution of female preferences and male mating signals.

## Introduction

Many theoretical models of sexual selection assume the existence of considerable, additive and correlated genetic variation for both the preferred male trait and female mating preference within populations [1]–[4]. Accordingly, examples of quantified variance in female mating preferences and their co-variance with male traits have been reported by several researchers [5]–[9], but not by others [10]–[12]. A genetic co-variance between preference traits and sexual traits may arise through assortative mating generating non-random associations between alleles at different loci controlling these traits (linkage disequilibrium) [13], [14]. Moreover, genetic associations may be affected either by genes influencing both traits that are located nearby within a chromosomal region (physical linkage) [14] or by genes coding for the expression of both traits (pleiotropy) [14], [15]. While several genetic studies provide support for a co-variance based on linkage disequilibrium (e.g. [7], [16], [17]), empirical evidence for a genetic coupling of both traits based on pleiotropy is comparably scarce (but see [18], [19]). Nevertheless, since genetic linkage between mating preferences and preferred traits can shield genetic co-variance from recombination as long as females can choose mates according to their preferences, pleiotropy and physical linkage may in some cases strongly contribute to the co-evolution of sexually selected traits. The identification of potential targets of selection, especially a precise characterization of mating preferences, may enhance understanding the processes involved in trait elaboration [8], [20].

Mating preferences comprise the entire set of sensory and behavioral characters which lead to a bias in mating decisions and are determined by both preference functions, defined as the ranking order of stimuli, and choosiness, defined as the effort in mate assessment [21]. Individual variation in mating preferences can be influenced by condition [22]–[25], age [26], experience [27], [28], search costs [29], genotype by environment interactions [30], [31], genetic compatibility [32] or the assessment of multiple traits [33]–[35]. Alternatively, among-individual variability in genetic predispositions can simply be expressed in phenotypic differences in the sensory apparatus which may, for example, result in different perceptual and discriminatory abilities in females [21]. However, the proximate basis of variation in female preference functions is rarely explored [36], [37] although, as previously depicted, reliable knowledge of the underlying mechanisms should provide useful information on the evolution of sexually selected traits. For instance, the sensory drive hypothesis addresses the mechanistic basis of mating preferences in that it predicts that females prefer a specific male signal design which maximally stimulates their sensory system and is thus more conspicuous and easier to detect in their local environment [38]–[40]. Consequently, studying the role of sensory perception in mate choice may further help in identifying the processes that promote divergence in sexual signals and preferences, resulting in reproductive isolation.

Much of the empirical work investigating sexual selection has focused on the important role of conspicuous visual signals in mate attraction in various taxa [41]. For example, the characteristic carotenoid-based red throat coloration of breeding male threespine sticklebacks (*Gasterosteus aculeatus*) is one of the best studied color signals in nature [42] which has provided important insights into intersexual [43] and intrasexual selection [44] as well as speciation [45]. Sticklebacks are capable of responding to visual signals incorporating wavelengths ranging from the ultraviolet (UV) to the ‘red’ part of the spectrum due to four retinal cone receptor types (UV, S, M, L) with cone absorbance maxima at around 360, 445, 530, and 605 nm, respectively [46]. In most populations female sticklebacks show a visual preference for mating with males that develop a greater intensity and extent of red coloration [47]. The degree of red coloration in males shows large variation [48], [49] and appears to signal both direct [50] and indirect benefits [5], [43] to females. Accordingly, stickleback males fed with lower levels of dietary carotenoids cannot maintain their red coloration and suffer more from oxidative damage due to the dual function of carotenoids as sexual signals and antioxidants [51].

By referring to the genetic basis of mate choice evolution in sticklebacks, Bakker [5] found a positive genetic correlation between female preference for male red coloration and red color expression in males on the intra-population level. Hence, in order to shed light on the proximate mechanisms underlying the co-evolution of male traits and female preferences we tested here if there is a visual component potentially accounting for among-female variability in mating preferences and whether it is associated with male color expression. To do this, we compared the intensity of the red nuptial coloration in stickleback males with their daughters' visual sensitivity to orange-red wavelengths, which was measured as optomotor response behavior.

A direct association between visual sensitivity and female mating preferences is largely unknown. Nevertheless, several studies suggested a causal relationship between visual perception and mating decisions at the level of species divergence [36], [52], [53]. We thus propose that variation in visual sensitivity in females might contribute to mating preferences since an improved visual perception should promote detection of male red coloration as well as discrimination between varying degrees of male coloration.

## Materials and Methods

### Ethics statement

Our study adhered to the Association for the Study of Animal Behaviour's Guidelines for the Use of Animals in Research and was carried out according to the German laws for experimentation with animals (§ 8 Abs. 1 TierSchG, V.m. § 2 Abs. 1.1 TierSchZustV NW 26.9.1989). No additional licences were required for performing non-invasive experiments with fish. After the study, all fish were kept in the laboratory as breeding stock for future experiments.

### Animal collection and maintenance

Threespine sticklebacks from an anadromous, genetically heterogeneous population [54] were caught during spring migration in April 2008 on the island of Texel, The Netherlands. In the laboratory, reproductively active males were individually moved to holding aquaria equipped with nesting material. Males were fed daily with *Chironomus* spp. in excess. Ripe females were visually presented to the males to induce nest-building. After nest completion males were paired with randomly sampled females from the same population to generate unrelated full-sib families. Two hours after fertilization male coloration was quantified spectrophotometrically (see below) and eggs were removed to exclude paternal effects on offspring traits. Progeny was raised artificially in full-sibling groups under standardized laboratory conditions until sexual maturation. Individuals were fed daily with *Artemia nauplii* during the first month of age and with *Chironomus* spp. in excess later on. At an age of about 20 months reproductively active females from the F1 generation (one female from each family) were then randomly sampled from the full-sib groups in order to use them in optomotor tests to measure their spectral sensitivity (see below).

### Measurement of male coloration

Standardized reflectance scans of each male were recorded with a spectrophotometer (Avantes AVS-USB2000) connected to a deuterium-halogen light source (Avantes DH-2000) for illumination. A bifurcated 200-µm fibre-optic probe with unidirectional illumination and recording was held at a 90° angle to the body surface with the probe end being inserted in a darkened pipette tip in order to exclude ambient light and to take measurements at a fixed distance of 0.3 cm from the surface. In order to eliminate measurement errors caused by body movements, males were quickly sacrificed by decapitation and then immediately placed on a piece of black fabric. Scans were collected from the orange-red cheek region below the eye. Reflectance intensity was measured relative to a 98% Spectralon white standard over the range of 300–700 nm at about 0.5-nm resolution in wavelength. Data were recorded with Spectrawin 5.1 (Avantes) and imported into Microsoft Excel. Fifteen measurements were made in succession averaged for the sample region without changing probe contact. The whole procedure took about one minute so that postmortem color changes due to either pigment aggregation or dispersion could be ruled out (IPR, personal observation).

The double-peaked nature of stickleback male cheek reflectance [55] is difficult to interpret in terms of chromatic variables [56]. We thus analyzed male orange-red coloration using two complementary approaches. We first quantified spectral purity of orange-red coloration from the reflectance data by computing the colorimetric variable ‘red chroma’ as the amount of light reflected in the range of 575–700 nm relative to the total amount of light in the range of 300–700 nm [57] taking into account the approximate visible spectrum of sticklebacks including ultraviolet (UV) wavelengths [46].

In addition, we used a physiological model on stickleback vision in order to quantify male red coloration as viewed through the female visual system. Therefore, spectral sensitivity curves for the four stickleback cone receptors were determined from cone absorbance maxima and based on a vitamin A2 based visual pigment template [46] by using parameters provided in Govardovskii et al. [58]. In the absence of detailed information on chromophore usage for sticklebacks from our study population we assumed the presence of a porphyropsin-dominated retina like it is commonly found in anadromous teleost species during the reproductive phase in freshwater habitats (e.g. [59]).

**Figure 1:**
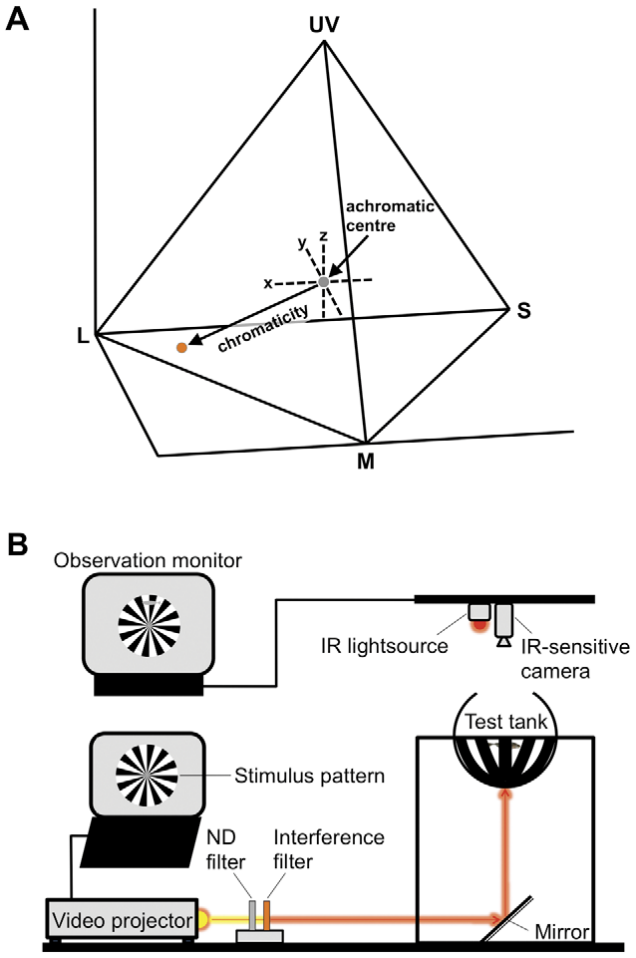
Methods used for measurement of male red coloration and female red sensitivity. (A) Color tetrahedron based on stickleback spectral sensitivity functions with each of the four corner points representing the exclusive excitation of a single cone (UV, S, M, L). The three independent coordinates x, y and z define the position of a spectral color in the three-dimensional space. Chromaticity was determined as the distance between a measured color point (orange dot) and the achromatic origin (grey dot) and represents the degree of chromatic difference between both locations. (B) Schematic representation of the optomotor setup used to measure visual sensitivity of female threespine sticklebacks. Test fish were exposed to a rotating stimulus pattern under three different stimulus wavelengths in the orange-red spectral region (590, 610 and 630 nm) generated by interference filters. To determine threshold sensitivity levels light intensity was increased in five steps by using a series of ND filters (see text for details).

We then calculated absolute quantum catch values for each cone receptor (UV, S, M, L) by multiplying spectral reflectance of the red cheek region per individual male by the spectral sensitivity of the cones and the ambient irradiance spectrum (standard daylight illuminant D65) between 300 and 700 nm [60]. The fish used in our study derived from clear and shallow waters and communicate over short distances so that we did not include absorbance and scatter by water in our computations. Furthermore, lens transmission properties can be neglected for the spectral range considered here (IPR, unpublished data). Absolute quantum catches for the four single cones were converted to relative quantum catches (Q_UV_, Q_S_, Q_M_, Q_L_) by dividing excitation of each cone by the sum of excitations for all four cone classes (e.g. Q_L_ = L/(UV+S+M+L)). From these relative quantum catches we computed Cartesian coordinates in tetrahedral color space (x, y, z) based on formulae provided by Kelber et al. ([61]; Fig. 1A). We then determined chromaticity as an estimate of intensity of the carotenoid-based orange-red coloration which is calculated as the Euclidean distance to the achromatic center (equal stimulation of all cones) [62].

### Measurement of female sensitivity

One day before sensitivity measurements, ripe females, as assessed by their distended abdomen and cloaca, were individually placed into holding aquaria. Female perceptual sensitivity was then estimated with the optomotor response technique, which has been successfully used to measure stickleback visual perception before [45], [63], [64]. Therefore, a pattern of 20 alternating black and white radial sectors arranged in a disk and rotating at 10 rpm was generated in Microsoft Powerpoint and projected from a digital video projector with adjusted gamma correction settings (Hitachi CP-X1200), via a mirror, on the lower half of a spherical opal glass lampshade, 25 cm in diameter, that served as the experimental tank (Fig. 1B). Fish were kept in a light-adapted state for five minutes prior to testing and for one minute between stimulus presentations by projecting a full white stimulus reduced to an intensity of 60% (275 lx) using a combination of neutral density filters (ND filters, Cotech). The spectral content of the stimulus pattern was controlled with three narrow band interference filters (590, 610 and 630 nm, Eureca Messtechnik), which were presented to the test fish in random order. To determine threshold sensitivity levels, light intensity was increased using a series of five neutral density filters (ND-filters, Andover Corporation). We chose a stepwise increase in light intensity instead of a decrease because preliminary trials testing both directions indicated that the former allowed for a better differentiation between nondirectional and directional swimming behavior of the test fish. Fifteen trials were performed per rotation direction with each trial lasting for one minute and being alternated with a one-minute period of adaptation light. The second half of the experiment was performed in an analogous manner but with reversed stimulus rotation. Behavior of the test fish was visualized and monitored using an infrared (IR) lightsource (Security-Center TV6700) combined with an infrared (IR) sensitive CCD camera (Everfocus CCIR) placed above the setup. We quantified the ‘optomotor gain’ by calculating the difference between clockwise and counter-clockwise pattern movement of the test female, divided it by the number of rotations of the pattern within one minute and calculated the mean of both pattern directions [65]. The relative sensitivity was determined for each test wavelength as the minimal light intensity at which an optomotor gain of 0.3 was reached in proportion to the lowest overall light intensity (darkest ND filter). The three interference filters differed slightly in quantal flux as revealed by spectrophotometric measurements of light intensity in the experimental setup with the detector probe placed in the center of the sphere and directed towards the striped pattern. Hence, filter transmission was balanced by combining interference filters with additional neutral density filters (ND filters, Cotech).

### Statistical analyses

Parametric statistics were used throughout the data analysis since data did not significantly deviate from normal distribution according to Shapiro Wilk tests. To reach normality, a negative reciprocal transformation was applied to the variable red chroma. Relative sensitivity of daughters between test wavelengths was compared using paired t-tests. Linear regressions were performed on both color variables of fathers (red chroma, chromaticity) versus the relative sensitivity of daughters for each separate test wavelength. Analyses were conducted using SPSS 12. All given *P*-values were based on two tailed-tests.

## Results

### Male coloration

**Figure 2:**
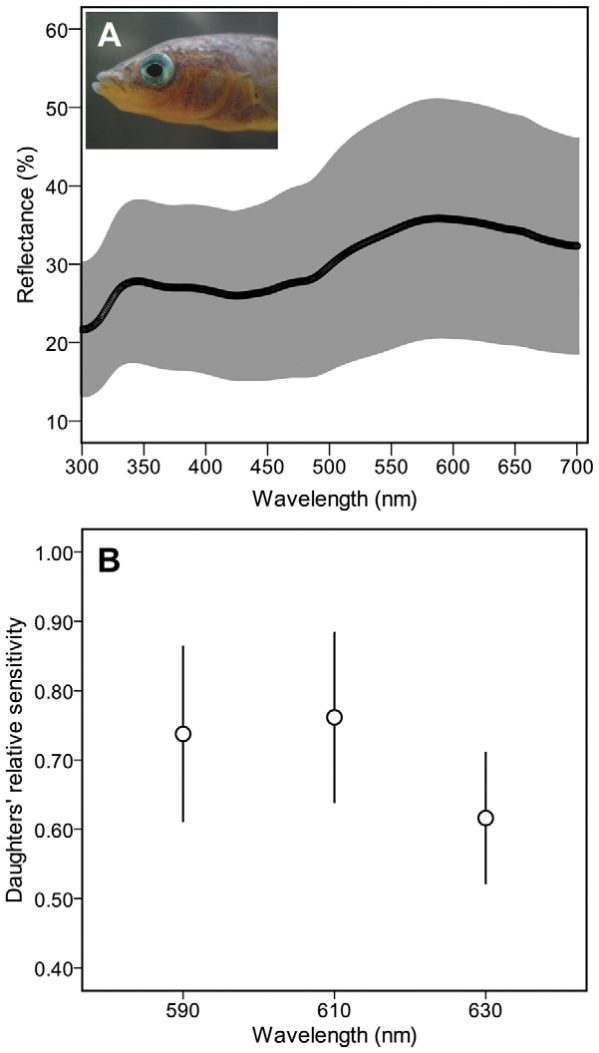
Spectral data on male red coloration and female red sensitivity. (A) Spectral reflectance for the cheek region of 25 reproductively active males. Plotted is the mean of the reflectance intensities (black line) ± standard deviation of the mean (shaded area). Reflectance was measured relative to a 98% white reference standard. (B) Mean relative sensitivity of daughters at three wavelengths of long-wave light (590, 610 and 630 nm) as measured in optomotor response tests. Error bars indicate standard deviation of the mean.

Reflectance spectra of the red-colored cheek region revealed distinct inter-male variation across the 300–700 nm waveband and showed a characteristic bimodal pattern of reflectance consisting of a major reflectance band at longer visible wavelengths (500–700 nm), a secondary peak in the near UV (300–400 nm), and a major absorption band at intervening wavelengths (400–500 nm). This is due to the absorptive properties of carotenoid pigments between 400 and 500 nm in combination with broadband reflectance of the underlying structural coloration ([66], [67]; Fig. 2A).

### Female sensitivity

We found substantial variation in spectral sensitivity of daughters towards visual stimuli in the orange-red part of the spectrum (Fig. 2B). Furthermore, relative sensitivity varied between the 590 nm and 630 nm test wavelengths (*t*_24_ = 6.440, P<0.001) as well as between the 610 nm and 630 nm wavelengths (*t*_24_ = 5.797, P<0.001) but not between the 590 nm and 610 nm wavelengths (*t*_24_ = −1.259, P = 0.220), analogous to results from previous optomotor tests on sticklebacks [63], [64], with higher values for the 590 nm and 610 nm steps that were closest to the absorbance maximum of the stickleback longwave-sensitive cone visual pigment [46].

### Father-daughter comparison

**Figure 3:**
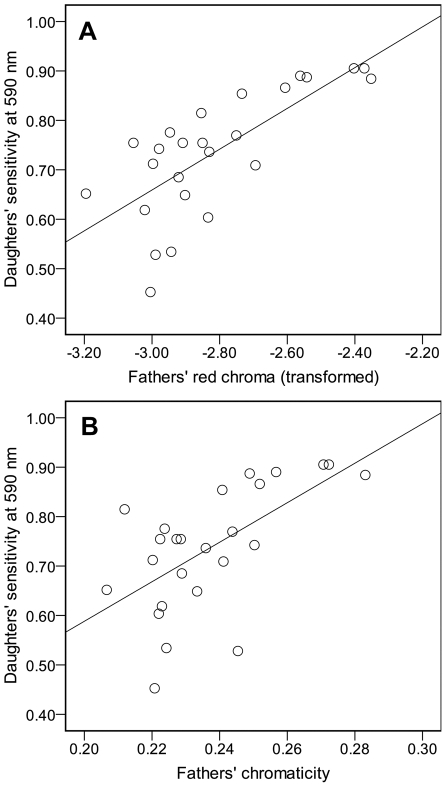
Comparison between red color expression in fathers and the red sensitivity of their daughters (n = 25). Relationship between daughters' relative sensitivity at 590 nm and fathers' (A) red chroma (after negative reciprocal transformation) and (B) chromaticity, respectively. The lines are the least square regressions [(A): *Y* = 0.413x+1.897, *R* ^2^ = 0.547, *F* = 27.80, *P*<0.0001; (B): *Y* = 3.997x−0.211, *R* ^2^ = 0.373, *F* = 13.70, *P*<0.01].

Variation in red chroma of fathers correlated with variation in their daughters' relative sensitivity for 590 nm (Fig. 3A), 610 nm (*R^2^* = 0.216, *P*<0.05) and 630 nm (*R^2^* = 0.161, *P*<0.05). In addition, the fathers' chromaticity was positively related to their daughters' sensitivity for 590 nm (Fig. 3B) and, although not significantly, for 610 nm (*R^2^* = 0.123, *P* = 0.085) and 630 nm (*R^2^* = 0.145, *P* = 0.060). Taken together, these results suggest an association between receiver design in female sticklebacks and red color expression in males.

## Discussion

A tight coupling between male sexual signals and the sensory capabilities of females is a key mechanism influencing the direction of sexual selection as predicted by the sensory drive hypothesis [40] and has been demonstrated for various species [68], [69]. Accordingly, red sensitivity of stickleback females was found to increase with the onset of the breeding season thereby enhancing the efficacy of the visual system to detect courting males [64]. Moreover, inter-population differences in stickleback male nuptial coloration are tuned to female red sensitivity and both traits vary depending on environmental light conditions ultimately leading to reproductive isolation [45].

We found variation in female red sensitivity on the intra-population level potentially acting on female mating preferences. Individual variation in the structure and function of visual systems may arise from various factors such as differences in the developmental environment [70], [71] or from altered environmental conditions in mature individuals, which has been shown for the accumulation of diet-derived carotenoids in the retina of birds [72] or for a reduced visual sensitivity in carotenoid-deprived fruitflies [73]. Carotenoids are essential for visual perception in fish as well since they act as a major precursor to vitamin A, which derivative, retinal, forms the chromophoric group of photopigments [74]. Given that carotenoid pigments play an important role in the development of male nuptial coloration in sticklebacks [75], the association between red color intensity in fathers and red sensitivity in their daughters found in the present study may reflect a genetic basis of pigment allocation in skin chromatophores in males and in photopigment expression in females. Nevertheless, the physiological processes responsible for variation in visual perception in this species are largely unknown and need to be addressed at the receptoral and postreceptoral level by especially taking into account a potential key role of carotenoid pigments in tuning spectral sensitivity.

Theoretical modeling of color vision in another stickleback population suggests that perceived variation in male red coloration is largest at shorter wavelengths (<500 nm) based on an assumed color opponency between the longwave and shortwave cones [46]. Accordingly, variation in sensitivity to longer wavelengths as found in the present study might not account for an enhanced discriminatory ability of females among male red coloration but might rather improve overall detection and identification of nuptially colored males [46]. However, electrophysiological evidence for an opponent mechanism between the longwave and shortwave cone is lacking for sticklebacks. Furthermore, in the present study we did not refer to female perception at shorter wavelengths and due to potential differences in sensory and signaling characteristics between populations depending on light regime [45], [76] one cannot rule out that the variation between females in sensitivity to longer wavelengths shown here is accompanied by variation in the ability of females to discriminate among males differing in the degree of red coloration.

In general, our results give support for a within-population association between male ornamentation and female visual sensitivity. However, further work using a parent-offspring approach should include enhanced sample sizes and heritability estimates in order to provide a reliable estimate of the strength of a genetic correlation between both traits. Furthermore, since we did not address the direct association between visual perception and mating preferences more experimental data are needed in order to clarify whether variation in mating preferences is basically influenced by other more relevant factors (see introduction) instead of red sensitivity alone. Moreover, from our results one cannot conclude whether females simply vary in red sensitivity or in overall visual perception, which should be adressed in future research covering the whole range of potential stimulus wavelengths.

Nevertheless, since female preference for red could be mediated by inter-individual variation in red perception our findings suggest that a sensory mechanism in terms of visual sensitivity is involved in the genetic correlation between female preference and the preferred male trait in this species. The potential dual function of carotenoids in both vision and signaling make pleiotropy a possible cause for the association found here. Such a genetic coupling was suggested by recent molecular genetic studies [8], [18], [19], [77] for different sensory modalities. The observed co-variance may also be due to linkage disequilibrium for loci affecting male nuptial coloration and female visual sensitivity maintained by assortative mating. However, in linkage disequilibrium the genetic correlation between unlinked genes is reduced by 50% after each generation of random mating due to recombination while it will decline to a lesser extent under physical linkage [14]. Since mate choice was prevented in the present study by forced random pairings for one generation the observed father-daughter association may rather indicate an influence of physical linkage or pleiotropy. Nonetheless, distinguishing between these mechanisms is difficult since the underlying genetic structure of our study population is unknown. Recombination-based genomic approaches such as linkage mapping for the identification of genes and genetic regions underlying signal production in stickleback males as well as female sensitivity thresholds may improve understanding the association found here within the overall context of ornament and mating preference co-evolution.
